# Clinical Phenotype of Graves Disease in Autoimmune Polyglandular Syndrome or as Isolated Disease: The GRAPHE Study

**DOI:** 10.1210/clinem/dgaf144

**Published:** 2025-03-01

**Authors:** Elisa Gatta, Ilenia Pirola, Aurora Gotti, Micaela Fredi, Pietro Bellini, Francesco Dondi, Riccardo Morandi, Claudio Casella, Francesco Bertagna, Franco Franceschini, Mario Rotondi, Carlo Cappelli

**Affiliations:** Department of Clinical and Experimental Sciences, SSD Endocrinologia, University of Brescia, ASST Spedali Civili of Brescia, 25123, Brescia, Italy; Centro per la Diagnosi e Cura delle Neoplasie Endocrine e delle Malattie della Tiroide, University of Brescia, 25123, Brescia, Italy; Department of Clinical and Experimental Sciences, SSD Endocrinologia, University of Brescia, ASST Spedali Civili of Brescia, 25123, Brescia, Italy; Centro per la Diagnosi e Cura delle Neoplasie Endocrine e delle Malattie della Tiroide, University of Brescia, 25123, Brescia, Italy; Department of Clinical and Experimental Sciences, SSD Endocrinologia, University of Brescia, ASST Spedali Civili of Brescia, 25123, Brescia, Italy; Department of Clinical and Experimental Sciences, Rheumatology and Clinical Immunology, University of Brescia, ASST Spedali Civili of Brescia, 25123, Brescia, Italy; Nuclear Medicine, University of Brescia, ASST Spedali Civili, 25123, Brescia, Italy; Nuclear Medicine, University of Brescia, ASST Spedali Civili, 25123, Brescia, Italy; Centro per la Diagnosi e Cura delle Neoplasie Endocrine e delle Malattie della Tiroide, University of Brescia, 25123, Brescia, Italy; Department of Clinical and Experimental Sciences, Surgical Clinic, University of Brescia, ASST Spedali Civili, 25123, Brescia, Italy; Centro per la Diagnosi e Cura delle Neoplasie Endocrine e delle Malattie della Tiroide, University of Brescia, 25123, Brescia, Italy; Department of Clinical and Experimental Sciences, Surgical Clinic, University of Brescia, ASST Spedali Civili, 25123, Brescia, Italy; Centro per la Diagnosi e Cura delle Neoplasie Endocrine e delle Malattie della Tiroide, University of Brescia, 25123, Brescia, Italy; Nuclear Medicine, University of Brescia, ASST Spedali Civili, 25123, Brescia, Italy; Department of Clinical and Experimental Sciences, Rheumatology and Clinical Immunology, University of Brescia, ASST Spedali Civili of Brescia, 25123, Brescia, Italy; Istituti Clinici Scientifici Maugeri IRCCS, Unit of Endocrinology and Metabolism, Laboratory for Endocrine Disruptors, 27100, Pavia, Italy; Department of Internal Medicine and Therapeutics, University of Pavia, 27100, Pavia, Italy; Department of Clinical and Experimental Sciences, SSD Endocrinologia, University of Brescia, ASST Spedali Civili of Brescia, 25123, Brescia, Italy; Centro per la Diagnosi e Cura delle Neoplasie Endocrine e delle Malattie della Tiroide, University of Brescia, 25123, Brescia, Italy

**Keywords:** Graves disease, autoimmune polyglandular syndrome, autoimmune diseases

## Abstract

**Context:**

Graves disease (GD) can occur as an isolated condition or as part of an autoimmune polyglandular syndrome; there are no data regarding the differences in phenotype between these 2 forms.

**Objective:**

To assess differences in clinical presentation, complications, and outcomes in patients with isolated GD compared to those in whom GD is part of an autoimmune polyglandular syndrome.

**Methods:**

The GRAPHE study is a retrospective observational study. The medical records of all patients with GD diagnosed and regularly followed at outpatient clinics for Endocrinology, Nuclear Medicine and Clinical Surgery, from January 1, 2010, to June 30, 2024, were retrieved. All the patients were followed by the same endocrinologists and treated in accordance with existing guidelines.

**Results:**

The enrolled patients (n = 567; 77% women) were divided into 3 different groups based on GD phenotypes: subjects affected by isolated GD (*isolated GD*); patients who developed autoimmune polyglandular syndrome (*GD1-APS*); and patients with autoimmune polyglandular syndrome who developed GD during follow-up (*GD2-APS*). The 3 groups were superimposable for gender (*P* = .086), fT4 (*P* = .899), fT3 (*P* = .434), TRAb titers (*P* = .882), and thyroid volume (*P* = .840) at disease onset. Isolated GD patients exhibited Graves orbitopathy more frequently (*P* < .001), a higher rate of definitive therapy (*P* < .001) and shorter time between disease onset and definitive therapy (*P* < .001) compared to the GD1-APS and GD2-APS patients.

**Conclusion:**

The results of the present study show that, despite similar clinical and biochemical phenotype at presentation, a more severe clinical course characterizes isolated GD patients compared to those whose disease is a feature of autoimmune polyglandular syndrome.

Graves disease (GD) is an autoimmune disease characterized by hyperthyroidism, goiter, thyroid eye disease (also referred to as Graves orbitopathy [GO]), and, less commonly, dermopathy (1). It represents the most common cause of hyperthyroidism (2), with a higher prevalence in women (ratio 5-10:1) and a incidence peak between 30 and 50 years of age (3). The overall prevalence is about 1.2% worldwide (4).

GD is caused by autoantibodies to the thyrotropin (TSH) receptor (TRAb) which stimulate thyroid hormone synthesis and secretion as well as thyroid growth (5). In addition, TRAb can bind TSH receptor on orbital fibroblasts, inducing glycosaminoglycans production and promoting their transformation into adipocytes, thus leading to GO manifestations (6-8). Approximately 25% to 50% of patients with GD had clinically detectable eye involvement (9, 10), with 6% of subjects affected by moderate to severe GO (9).

First-line therapy for GD is pharmacological, with the use of thionamides aiming to control symptoms, reduce thyroid hormone production, and achieve remission of the disease. They inhibit the iodination of thyroglobulin mediated by thyroid peroxidase, thereby blocking the production of thyroxine (T4) and triiodothyronine (T3) (11). Despite adequate antithyroid treatment, relapse rates remain high, affecting more than 50% of cases, requiring definitive treatments (surgery or radioiodine), which are predominantly used in patients with moderate to severe GO (12).

Autoimmune polyglandular syndromes (APS) are rare disorders that encompass a wide range of autoimmune diseases, involving both endocrine and nonendocrine organs (13). The original classification divides APS into 4 groups; the subtypes characterized by the presence of autoimmune thyroid disease include APS type 2, which is defined by the presence of Addison disease associated with autoimmune thyroid disease and/or type 1 diabetes mellitus, and APS type 3, where autoimmune thyroid disease is associated with another autoimmune disorder (14). It is estimated that up to 30% of patients with autoimmune thyroid disease either have or will develop a second autoimmune disease during their lifetime, leading to a diagnosis of APS (15).

Recently, it has been described that patients with GD and other autoimmune diseases exhibit a higher rate of transition from hyperthyroidism to hypothyroidism compared to those with isolated GD (16).

In the present study, we investigated the clinical presentation, progression, complications, and outcomes of patients with isolated GD compared to those in whom GD is part of an APS.

## Methods

### Patients

We reviewed retrospectively the medical records of all patients with GD diagnosed and regularly followed at outpatient clinics for Endocrinology, Nuclear Medicine and Clinical Surgery, from January 1, 2010, to June 30, 2024. All the patients were followed by the same endocrinologists treating GD and GO in accordance with existing guidelines (12, 17, 18).

The study (ASST_BS_GRAPHE) was approved by the local Ethics Committee (no. 6323).

### Clinical Data Collection

The clinical records reviewed included all patients affected by GD. All patients were periodically screened for the most common autoimmune endocrinopathies, and for all autoimmune pathologies when clinically suspected, in accordance with the ORPHAcode (ORPHA 282196, 3453, 3143, 227982, 227990) (13). Demographic and anthropometric data were collected of GD diagnosis, TSH, free T4 (fT4), free T3 (fT3), and serum TRAb levels at diagnosis, type and dosage of antithyroid treatment, therapy and disease duration, other treatments taken, presence of GO, number and timing of disease relapses, and the need for and timing of definitive treatment; GO was defined in accordance with the European Group on Graves’ Orbitopathy (EUGOGO) clinical practice guidelines (17). All the data were collected anonymously in an electronic database. Patients with incomplete data were excluded from the present study.

### Statistical Analysis

Normal distribution was achieved using the Shapiro-Wilk test. Between-group comparison was performed using the Student *t* test for unpaired data or ANOVA for quantitative variables, as appropriate, and the χ^2^ test for categorical variables. A logistic regression analysis was performed to evaluate the impact of GD status (isolated GD, GD1-APS, or GD2-APS), sex, age at GD onset, fT4, fT3, TRAb levels, thyroid volume, and immunosuppressive therapy (independent variables) on the likelihood of receiving definitive treatment (categorized as binary outcome), adjusting for potential confounders. A *P* value <.05 was considered statistically significant. The statistical analyses were performed using SPSS 20.0 software (SPSS, Inc., Evanston, IL, USA).

The results are reported in compliance with the STROBE reporting guidelines for cross-sectional studies.

## Results

A total of 567 patients (77% women) affected by GD with a mean age of 50.4 ± 13.5 years were enrolled in the present study. The age at onset of GD was 41.9 ± 13.5 years, without difference between sex (41.6 ± 13.7 vs 43.4 ± 12.8, F/M; *P* = .521) with an average follow-up time of 16.7 ± 11.2 years. All the enrolled patients showed overt hyperthyroidism with a mean fT4 level of 28.5 ± 22.0 ng/L, fT3 level of 9.3 ± 7.2 ng/L, and a mean TRAb titer of 12.9 ± 19.5 IU/L. Among all the patients, 249 (44%) subjects experienced at least one GD relapse (42.9% vs 47.3%; F/M, *P* = .806), whereas GO developed in 167 (29.5%) cases (31.3% vs 28.9%, F/M; *P* = .253). Clinical activity score (CAS) ≥3 was observed in 20.5% of patients, while severe GO was present in 6.3%. Table 1 shows the clinical characteristics of the patients.

**Table 1.** Demographic and clinical data of the sample population

	All (567)	Isolated GD (N = 430)	APS (N = 137)	*P*
Male/female	131/436	103/327	28/109	.395
Age at GD onset, years	41.9 ± 13.5	42.6 ± 12.8	40.1 ± 15.4	.023
fT4, ng/L	28.5 ± 22.0	28.6 ± 22.7	27.0 ± 15.5	.052
fT3, ng/L	9.3 ± 7.2	9.4 ± 7.3	8.4 ± 6.6	.295
TRAb, UI/mL	12.9 ± 19.5	13.3 ± 20.6	11.5 ± 13.4	.918
Thyroid volume, mL	32 (14-96)	33 (14-99)	32 (13-87)	.763
Graves orbitopathy (GO)	29.5%	34.7%	15.7%	<.001
Active GO (CAS ≥ 3)	20.5%	24.9%	7.9%	.058
GO severity				
Mild	8.9%	8.8%	12.2%	<.001
Moderate	14.3%	18.3%	0.9%	
Severe	6.3%	7.6%	2.6%	
Definitive treatment	55.4%	66.0%	53.3%	.007
Time between disease onset and definitive therapy, years	4.0 ± 5.6	3.9 ± 4.1	8.6 ± 8.4	<.001

Abbreviations: APS, autoimmune polyglandular syndrome; CAS, clinical activity score; fT4, free thyroxine (normal value 9.3-17.0 ng/L); fT3, free triiodothyronine (normal value 2.0-4.4 ng/L); GD, Graves disease; TRAb, autoantibodies to the thyrotropin receptor (normal value <1.8 UI/mL).

**Figure 1.**
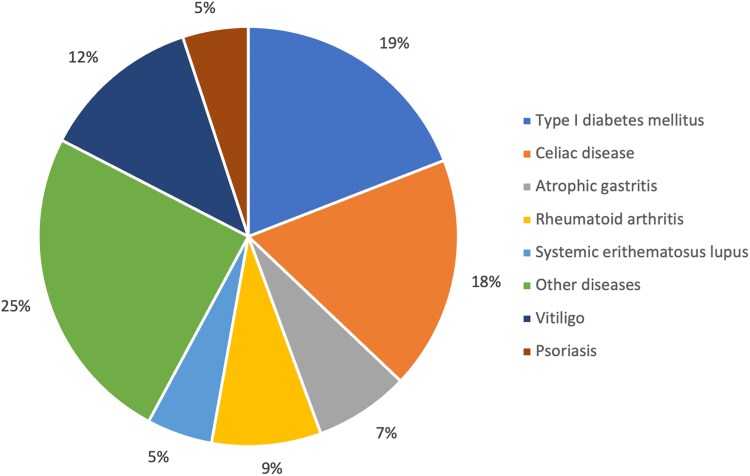
Prevalence of the various autoimmune conditions associated with GD in the APS group.

The patients were divided into 2 distinct groups based on GD phenotypes: those with GD who did not develop any other autoimmune disease during follow-up (isolated GD), and those with GD as a feature of APS (GD-APS). Figure 1 illustrates the prevalence of the various autoimmune conditions associated with GD in the APS group.

Patients with isolated GD developed GO more frequently than those with GD-APS (34.7% vs 15.7%, respectively; *P* < .001), with a higher proportion exhibiting severe GO (7.6% vs 2.6%, respectively; *P* < .001), although there was no significant difference in active GO (24.9% vs 7.9%, respectively; *P* = .058). Isolated GD patients also had a higher rate of definitive therapy (66% vs 53.3%; *P* = .007). Additionally, the time between disease onset and definitive therapy was significantly shorter in the isolated GD group (3.9 ± 4.1 years) compared to the GD-APS group (8.6 ± 8.4 years; *P* < .001). No other demographic or clinical phenotypic differences were observed (Table 1).

To further investigate the role of APS, GD-APS patients were subsequently divided into 2 groups: patients with GD who developed APS (GD1-APS), and patients with APS who developed GD during follow-up (GD2-APS). Table 2 presents demographic and clinical characteristics of the 3 groups. In detail, 430 subjects (76%) were included in the isolated GD group, 48 (8%) in the GD1-APS group, and 89 (16%) in the GD2-APS group. The groups of patients were comparable in terms of gender distribution (*P* = .086), mean fT4 levels (*P* = .899), fT3 levels (*P* = .434), TRAb titers (*P* = .882), and thyroid volume (*P* = .840) at diagnosis. GO occurred significantly more frequently in the isolated GD group (34.7%) compared to in the GD1-APS and GD2-APS patients (23.7% and 11.7%, respectively, *P* < .001). In addition, patients in the isolated GD group displayed a higher rate of definitive therapy (66% vs 39.6% vs 60.7%, isolated GD vs GD1-APS vs GD2-APS; *P* < .001). The time between disease onset and definitive therapy was significantly shorter in the isolated GD group (3.9 ± 4.1 years) compared to the GD1-APS (5.0 ± 6.9 years) and GD2-APS (9.2 ± .6 years) groups (*P* < .001).

**Table 2.** Demographic and clinical data of the subject sample divided by groups

	Isolated GD (N = 430)	GD1-APS (N = 48)	GD2-APS (N = 89)	*P*
Male/female	103/327	5/43	23/66	.086
Age at GD onset, years	42.6 ± 12.8	32.5 ± 12.1	44.2 ± 15.6	<.001
fT4, ng/L	28.6 ± 22.7	32.2 ± 13.4	26.8 ± 15.7	.899
fT3, ng/L	9.4 ± 7.3	8.0 ± 7.3	6.9 ± 3.4	.434
TRAb, UI/mL	13.3 ± 20.6	12.3 ± 13.8	11.6 ± 13.1	.822
Thyroid volume, mL	33 (14-99)	27 (15-78)	34 (11-99)	.840
Graves orbitopathy (GO)	34.7%	23.7%	11.7%	<.001
Active GO (CAS ≥ 3)	24.9%	10.5%	6.5%	.145
GO severity				
Mild	8.8%	18.4%	9.1%	<.001
Moderate	18.3%	1.3%	1.3%	
Severe	7.6%	3.9%	1.3%	
Definitive treatment	66.0%	39.6%	60.7%	.001
Time between disease onset and definitive therapy, years	3.9 ± 4.1	5.0 ± 6.9	9.2 ± 0.6	<.001

Abbreviations: CAS, clinical activity score; fT4, free thyroxine (normal value 9.3-17.0 ng/L); fT3, free triiodothyronine (normal value 2.0-4.4 ng/L); GD, Graves disease; GD1-APS, patients with GD who developed APS; GD2-APS, patients with APS who developed GD; TRAb, autoantibodies to the thyrotropin receptor (normal value <1.8 UI/mL).

To exclude the potential interference of immunosuppressive therapy, a subanalysis was performed, excluding patients who had undergone or were undergoing treatment with immunomodulatory drugs throughout the study span. A total of 517 patients (76% female) with a mean age of 49.8 ± 13.1 years were included in the subanalysis. Specifically, 430 (83%) subjects were in the isolated GD group, 34 (7%) in the GD1-APS group, and 53 (10%) in the GD2-APS group. The 3 groups were comparable in terms of gender distribution (*P* = .416), mean fT4 levels (*P* = .652), fT3 levels (*P* = .244), TRAb titers (*P* = .433), and thyroid volume (*P* = .863) at diagnosis. GO occurred significantly more frequently in the isolated GD group (34.7%) compared to in the GD1-APS patients (25%; *P* < .001) and GD2-APS patients (8%, *P* = .003). Patients in the isolated GD group had a significantly lower remission rate compared to the other groups (66% vs 47.1% vs 58.5%, isolated vs GD1-APS vs GD2-APS, *P* = .05) and shorter time between disease onset and definitive therapy (3.9 ± 4.1 years vs 5.3 ± 6.0 and 8.2 ± 8.3, isolated vs GD1-APS vs GD2-APS; *P* < .001). Table 3 summarizes the demographic and clinical data of the patients.

**Table 3.** Demographic and clinical data of the subjects not undergoing immunosuppressive therapy, divided by groups

	Isolated GD (N = 430)	GD1-APS (N = 34)	GD2-APS (N = 53)	*P*
Male/female	103/327	5/29	14/39	.416
Age at GD onset, years	42.6 ± 12.8	32.1 ± 10.3	44.9 ± 15.1	<.001
fT4, ng/L	28.6 ± 22.7	27.4 ± 19.6	26.2 ± 15.7	.652
fT3, ng/L	9.4 ± 7.3	7.8 ± 4.1	6.7 ± 3.2	.244
TRAb, UI/mL	13.3 ± 20.6	8.7 ± 13.9	10.2 ± 12.0	.822
Thyroid volume, mL	33 (14-99)	28 (15-76)	21 (11-96)	.863
Graves orbitopathy	34.7%	25%	8.0%	.003
Definitive treatment	66.0%	47.1%	58.5%	.05
Time between disease onset and definitive therapy, years	3.9 ± 4.1	5.3 ± 6.0	8.2 ± 8.3	<.001

Abbreviations: fT4, free thyroxine (normal value 9.3-17.0 ng/L); fT3, free triiodothyronine (normal value 2.0-4.4 ng/L); GD, Graves disease; GD1-APS, patients with GD who developed APS; GD2-APS, patients with APS who developed GD; TRAb, autoantibodies to the thyrotropin receptor (normal value <1.8 UI/mL).

A logistic regression analysis, which included isolated GD, GD1-APS, and GD2-APS patients, along with age at GD onset, sex, fT4, fT3, TRAb titers, thyroid volume, and immunosuppressive therapy, identified the GD phenotype (particularly isolated GD) as the only predictor of the need for definitive treatment (OR 10.743, 95% CI 1.484-77.760; *P* = .019) (Table 4).

**Table 4.** Logistic regression analysis of definitive treatment predictors in the study population

	OR (CI 95%)	*P*
GD1-APS	—	.063
GD2-APS	—	1
Isolated GD	10.743 (1.484-77.760)	.019
Sex	1.209 (0.224-6.533)	.825
Age at GD onset, years	1.018 (0.972-1.065)	.451
fT4, ng/L	1.029 (0.972-1.089)	.325
fT3, ng/L	1.016 (0.887-1.163)	.819
TRAb, UI/mL	1.054 (0.983-1.129)	.138
Thyroid volume	3.447 (0.757-18.142)	.160
Immunosuppressive therapy	0.225 (0.012-4.270)	.320

Abbreviations: fT4, free thyroxine (normal value 9.3-17.0 ng/L); fT3, free triiodothyronine (normal value 2.0-4.4 ng/L); GD, Graves disease; GD1-APS, patients with GD who developed APS; GD2-APS, patients with APS who developed GD; OR, odds ratio; TRAb, autoantibodies to the thyrotropin receptor (normal value <1.8 UI/mL).

## Discussion

Our study showed that at disease onset, isolated GD patients and those with GD in APS exhibited similar clinical and biochemical phenotype, as the 3 groups of patients (isolated GD, GD1-APS, and GD2-APS) were superimposable for TSH, fT4, and fT3 serum levels, TRAb titers, and thyroid volume. However, despite the above similarities, patients with isolated GD exhibit a more severe clinical course of GD as compared to those whose disease is a feature of APS in terms of: (i) higher prevalence of GO; (ii) higher rate of patients addressed to definitive therapy; and (iii) shorter time between disease onset and definitive therapy. First, even if the prevalence of GO in isolated GD (35%) was in line with literature (25%-40%) (9), it was significantly higher (*P* < .001) compared with patients with APS (Table 2). Second, the isolated GD patients required definitive ablative treatment, most commonly thyroidectomy, in 66% of cases, compared to 40% in GD1-APS and 60% in GD2-APS patients (*P* < .001). Another indicator of disease aggressiveness was the time between GD onset and the need for definitive treatment. Once again, definitive treatment was required significantly earlier in isolated GD patients. It became necessary, after a mean time of 3.9 years from disease onset, compared to 5 years and 9.2 years in GD1-APS and GD2-APS patients, respectively. We must underline that selection and treatment bias were likely avoided since all patients were treated and followed by the same endocrinologists treating GD and GO in accordance with existing guidelines.

The subgroup analysis conducted to exclude the potential interference of immunosuppressive therapy reinforced our results: the data, in fact, confirmed that isolated GD patients were affected by a more aggressive phenotype for all the 3 items evaluated.

This analysis was needed, considering studies suggesting potential beneficial effects of immunomodulatory therapy on ocular complications, recurrences, and the need for definitive therapy in subjects affected by GD. Indeed, Struja et al showed in a recent meta-analysis a significant reduction in GD relapse in patients treated with systemic immunosuppressive therapy alongside antithyroid drugs (methimazole and/or propylthiouracil) (19).

Similarly, the recent clinical practice guidelines for the medical management of GO clearly underlined an improvement in the clinical prognosis of GO in patients concurrently treated with immunomodulatory drugs (azathioprine, mycophenolate mofetil, cyclosporine, and corticosteroids) (17).

Although the design of the present study would not allow drawing mechanistic conclusions as to the different phenotypes observed in isolated GD vs GD-APS, some recent evidence might be regarded as suggestive for a different activation and regulation pathways of the immune response between isolated GD patients and those affected by other autoimmune diseases (20). Indeed, very recently, Tompa and Faresjö observed a significant difference in B cell subsets between children with combined type 1 diabetes and celiac disease compared to those with exclusively one of these diseases (21). In particular, the authors highlighted an alteration in B cell subsets with an increased percentage of B cells and lower percentages of memory cells in patients with isolated disease (21). In addition, Wardermann et al showed that a majority (55%-75%) of all antibodies expressed by isolated B cells displayed self-reactivity (22). Thus, it could be speculated that the increased percentage of B cells in patients with isolated autoimmune diseases, such as our GD patients, may reflect a distinct and more aggressive phenotype.

Serum TRAb levels are the hallmark of GD and universally considered as a prognostic factor for the disease (21). High antibody titers are, in fact, correlated with a higher probability of developing GO and more severe disease (21, 22). Going on, the recent review by Hesargatta Shyamasunder and Abraham, evidenced that TRAb titer at disease onset can predict the prognosis (23). However, in the GRAPHE study, no significant differences were found in TRAb titers between isolated GD patients and those with APS, despite a different clinical course. Indeed, recently Rotondi et al suggested a possible explanation for the lack of correlation between disease aggressiveness and TRAb titers. The authors showed that patients with GD and other autoimmune diseases had markedly higher rates of transition from hyperthyroidism to hypothyroidism status than patients with isolated disease (16). It is well known that 3 types of TRAb with different functional behavior (stimulating, blocking, and neutral) exist and can coexist in GD patients (24, 25). This may explain how, in a minority of patients with GD, thyroid function can fluctuate in accordance with changes in the ratio of stimulating and blocking antibodies in their serum (26, 27). Unfortunately, our TRAb assay did not discriminate among the autoantibody types; consequently, we cannot prove that this was the explanation of our different GD phenotype. However, a high prevalence of blocking TRAb levels has been demonstrated in many autoimmune diseases, including in patients affected by rheumatoid arthritis (28). Our results cannot be fully explained without considering the genetic and epigenetic complexity and the immune pathways involved in the various autoimmune disease. Studies obtained in larger cohort of patients, using advanced TRAb assays and/or assessing B cell subsets are needed to clarify this important issue.

At present, the most reasonable hypothesis that could be drawn is that the immunological cascade involving multiple diseases may somehow be the cause or contributing factor in the shift from stimulating to blocking TRAb titers in addition to a lower immune response due to difference in B cell subsets.

## Data Availability

Original data generated and analyzed during this study are included in this published article.

## Abbreviations

APS: autoimmune polyglandular syndrome
fT3: free triiodothyronine
fT4: free thyroxine
GD: Graves disease
GD1-APS: patients with GD who developed APS
GD2-APS: patients with APS who developed GD
GO: Graves orbitopathy
TRAb: thyrotropin receptor autoantibody
TSH: thyrotropin (thyroid-stimulating hormone)
